# Physically informed data-driven modeling of active nematics

**DOI:** 10.1126/sciadv.abq6120

**Published:** 2023-07-05

**Authors:** Matthew Golden, Roman O. Grigoriev, Jyothishraj Nambisan, Alberto Fernandez-Nieves

**Affiliations:** ^1^ School of Physics, Georgia Institute of Technology, Atlanta, GA 30332, USA.; ^2^ Department of Condensed Matter Physics, University of Barcelona, Barcelona 08028, Spain.; ^3^ Institute of Complex Systems (UBICS), University of Barcelona, Barcelona 08028, Spain.; ^4^ ICREA-Institució Catalanade Recerca i Estudis Avançats, Barcelona 08010, Spain.

## Abstract

A continuum description is essential for understanding a variety of collective phenomena in active matter. However, building quantitative continuum models of active matter from first principles can be extremely challenging due to both the gaps in our knowledge and the complicated structure of nonlinear interactions. Here, we use a physically informed data-driven approach to construct a complete mathematical model of an active nematic from experimental data describing kinesin-driven microtubule bundles confined to an oil-water interface. We find that the structure of the model is similar to the Leslie-Ericksen and Beris-Edwards models, but there are appreciable and important differences. Rather unexpectedly, elastic effects are found to play no role in the experiments considered, with the dynamics controlled entirely by the balance between active stresses and friction stresses.

## Teaser

A physics-informed data driven method constructs a complete model of an experimental active nematic system.

## INTRODUCTION

Active matter and the associated emergent phenomena such as spontaneous organized motion have attracted a lot of attention recently (*1*–*5*). Many different types of active matter exist at different length scales. One particularly interesting and common type is active nematics (*6*), which feature highly elongated apolar interacting units. Some notable examples include systems composed of vibrated monolayers of cylindrical rods (*7*), microtubules (*8*), actin filaments (*9*), and certain types of bacteria (*10*) suspended in a layer of fluid.

Our current theoretical understanding of active nematics is mainly based on the Leslie-Ericksen model (*11*, *12*) and the related Beris-Edwards model (*13*, *14*), which both provide a coarse-grained description of microscopic nematic molecules in three spatial dimensions. Recent interest in active nematics has been spurred by experimental studies most of which used a similar experimental setup where a suspension of microtubules is confined at a flat interface between a layer of water and a layer of oil (*8*, *15*–*17*). This quasi–two-dimensional system exhibits a range of complex defect-mediated flows. A number of hydrodynamic models—all versions of either Leslie-Ericksen or Beris-Edwards model—have been proposed to understand and quantify the observed flow patterns and transitions between dynamical regimes (*6*, *18*–*27*). While these models are capable of capturing some qualitative features of experimental flows (*9*, *28*–*32*), they also fail to predict or explain some observations such as the onset of circular flow (*17*). These models also fail to reproduce the observed dependence of the characteristic length scale on both activity (*16*) and surrounding-fluid viscosity (*33*).

A key difficulty in comparing theory with experiment for active nematics is that existing hydrodynamic models have a dozen or so parameters, few of which are measurable. Some progress has been made in indirectly identifying parameters in models of known functional form from experimental data for both filamentous bacteria (*10*) and microtubule suspensions (*34*). However, the correct functional form of the hydrodynamic model for a particular active nematic system remains undetermined. Furthermore, theoretical analyses uniformly assume, but never prove, that the system can be described by a two-dimensional model, whatever its functional form. As mentioned previously, the Leslie-Ericksen and Beris-Edwards models have been derived for three-dimensional suspensions of microscopic nematics and have no first-principles justification for macroscopic microtubule bundles confined to a two-dimensional interface between a pair of fluid layers. The presence of these fluid layers, which are themselves in contact with one or more rigid boundaries, is rarely taken into account (*33*). Yet, the flow in these layers, which contain no active nematic, and confinement by the rigid boundaries can play a nontrivial role that extends well beyond generating Rayleigh friction (*35*, *36*). While the motion of microtubules is confined to the two-dimensional interface, the flow in both fluid layers driven by that motion generally remains fully three-dimensional. While both fluids are incompressible in three dimensions, the interfacial flow generally will not satisfy the divergence-free conditions in two spatial dimensions as is usually assumed.

The objectives of the present study are therefore to (i) determine whether the dynamics of microtubule suspensions confined to a two-dimensional interface can indeed be described by a two-dimensional model, (ii) determine the functional form of the model, and (iii) determine the values of all the coefficients from experimental data. To achieve these objectives, we will introduce an algorithm known as sparse physics-informed discovery of empiric relations (SPIDER). Building on a body of machine learning literature devoted to spatially extended nonequilibrium systems (*37*–*43*), SPIDER combines the relevant domain knowledge and experimental measurements to identify a complete set of parsimonious physical relations necessary to describe the observed dynamics quantitatively. Unlike neural networks that can be trained to reproduce the observed dynamics, including those of active nematics (*34*, *44*) but yield little physical insight, SPIDER is designed to generate a set of physical relations in the familiar form of partial differential equations (PDEs) which can be directly compared against existing models. Moreover, these physical relations are easily interpretable and provide substantial physical insight, especially when discrepancies with existing models are found.

## RESULTS

### Data-driven model discovery

We will assume that a model of this quasi-two-dimensional active nematic system can be synthesized using measurements of three physical observables at the two-dimensional oil-water interface. These observables—the director field, the flow velocity, and a local order parameter—appear in most first-principles models of active nematics. In addition, we will avoid using numerous specific assumptions made by existing models and instead only use a few very general physical constraints, such as the symmetries of the problem, to make the analysis tractable.

Nematic symmetry **n** → −**n** combined with the presence of topological defects, which is a generic feature of this system, implies that the director field cannot be defined globally as a continuous field. To avoid this complication, most theoretical models instead use the globally continuous nematic tensor *Q_ij_* = *Sn_i_n_j_*, where 0 ≤ *S* ≤ 1 is a scalar order parameter, or its traceless counterpart ${\bar{Q}}_{ij}$. The scalar order parameter *S* measures the degree of local alignment of nematic molecules and mainly serves to describe disorder arising from thermal motion of microscopic nematic molecules. In contrast, away from defects, microtubules tend to be well-aligned due to a combination of their large aspect ratio and their relatively strong interaction; hence, we set *S* = 1 in our analysis.

Unlike molecular nematics, which tend to have a uniform density, microtubule-based systems are nonuniform, with their density or, more accurately, packing fraction ϕ varying between zero in the neighborhood of topological defects and unity far from the defects. The density field ϕ plays a role analogous—but not equivalent—to the order parameter *S*. Note that the values of ϕ ≈ 1 can only be achieved due the near-perfect alignment of the microtubules. While some phenomenological models that include the density field have been proposed (*19*, *45*), they lack proper justification or validation. Most commonly, ϕ is simply assumed to be a constant in the first-principles models of microtubule suspensions.

**Fig. 1.**
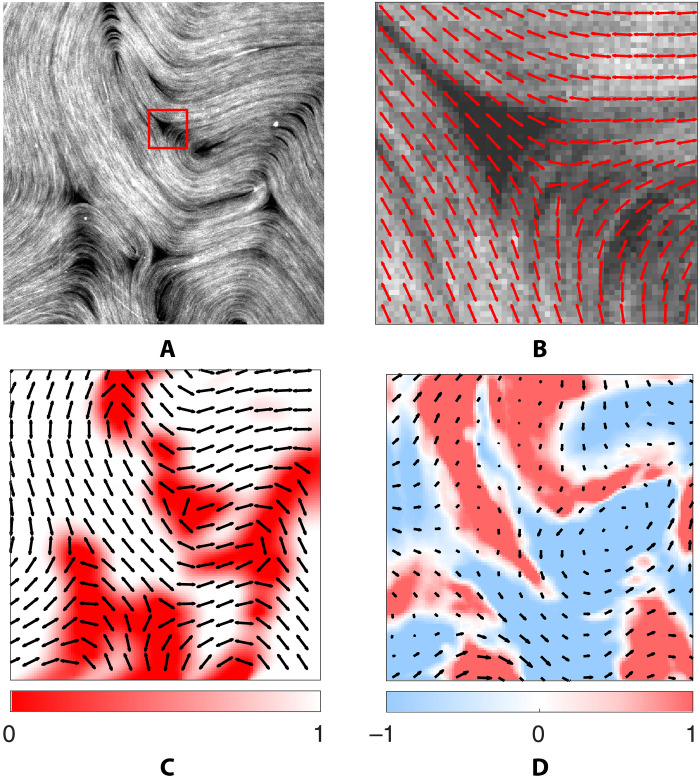
Raw experimental images and the extracted fields. (**A**) A snapshot of the microtubules. The complete image is shown, with the red box highlighting a small region centered on a −1/2 topological defect. **(B**) The zoomed-in view of the red box. The extracted director field **n** (red arrows) does not line up with the orientation of the microtubules in the center of the image, which indicates that director field data are unreliable near topological defects. (**C**) Director field (black arrows) and the mask ψ (color). (**D**) The flow field **u** (black arrows) and the corresponding vorticity ω = −2Ω*_xy_* (color). (C) and (D) The vector fields corresponding to (A) on a much coarser grid than that on which the data are available.

The director field **n**, the flow velocity **u**, and the microtubule density ϕ can be all extracted from sequences of snapshots of dense flourescently labeled microtubule bundles, such as the one shown in Fig. 1A. Examples of reconstructed fields are shown in Fig. 1 (B to D). The spatial resolution of the images however is insufficient to resolve the fast variation of ϕ and **n** near the topological defects. Moreover, no reliable information about either **n** or **u** can be obtained in the regions where ϕ ≈ 0 (dark areas in the image) which typically surround topological defects, as illustrated in Fig. 1B. As a result, we exclude these regions from our analysis. Outside of those regions, ϕ ≈ 1 is effectively constant. Therefore, only the fields **n** and **u** serve as useful data.

**Fig. 2.**
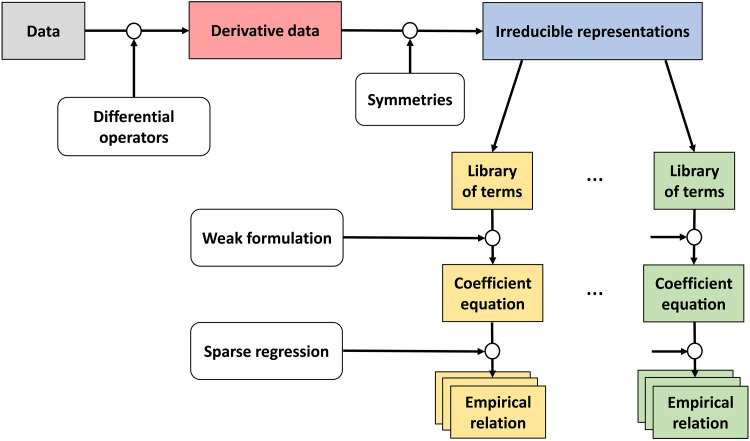
A schematic representation of the SPIDER algorithm. Tensors *F^r^* are constructed in symbolic form from fields and their derivatives and projected into irreducible representations of the underlying symmetry group, yielding a set of libraries. Weak form of the corresponding (Eq. 1) evaluated using appropriately sampled data yields a coefficient (Eq. 2). Last, a sparse regression algorithm is applied to each coefficient equation to identify one or more empirical relations.

These velocity and director fields are sufficient to synthesize a hydrodynamic model of this experimental system with the help of SPIDER, as described in detail in Methods. Figure 2 summarizes the key ingredients and steps of the algorithm. First, a sufficiently large set of tensor products *F^r^*(**n**, **u**), up to rank 2, is constructed in symbolic form from the vector fields **n** and **u** as well as their spatial and temporal derivatives. These are split into irreducible components according to the symmetries of the system, yielding libraries of terms with similar transformation laws. For instance, terms that are invariant with respect to the nematic symmetry and transform as vectors under rotation form one library, while terms that are invariant under rotations but change sign when **n** is replaced with −**n** form another. Each library represents a PDE of the form

$$\sum_{r}c_{r}F^{r}=0$$ (1)

with coefficients *c_r_* that are assumed to be constant, reflecting the symmetry of the problem with respect to translations in space and time. Note that the vertical confinement implies that any model that might be discovered would be two-dimensional, so $\nabla =\hat{x}\nabla_{x}+\hat{y}\nabla_{y}$, $u=\hat{x}u_{x}+\hat{y}u_{y}$, and so on.

To combat measurement noise (*41*), PDE (1) is converted to its weak form. Specifically, it is multiplied by one of several smooth weight functions and integrated over one of many rectangular spatiotemporal domains to generate an overdetermined linear system of equations

Gc=0 (2)

for the coefficients *c_r_*. The matrix *G* contains integrals of individual terms which are evaluated using properly nondimensionalized data. This process is repeated for each library, yielding a set of coefficient (Eq. 2). To make sure that regions with unreliable director and velocity field data are excluded, the weights are constructed as a product with a mask ψ that vanishes in the regions to be excluded. In practice, the mask is constructed automatically based on the values of ϕ and ∇*_i_n_j_*. An example of a mask overlaid on the director field is shown in Fig. 1C and corresponding movie S4, which shows how the mask evolves in time.

**Fig. 3.**
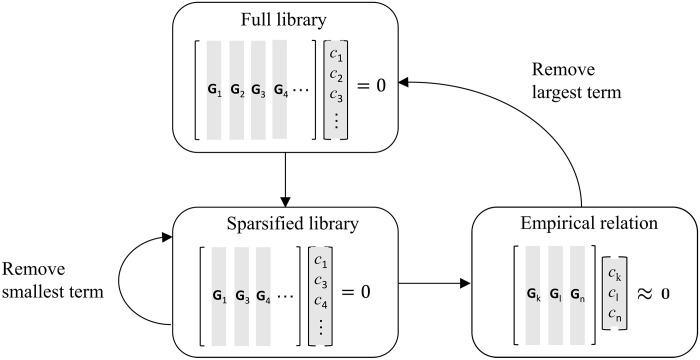
An iterative regression algorithm for finding a set of sparse solutions for the coefficient (Eq. 2). An empirical relation balancing sparsity and accuracy is identified using sequential regression of the full library. The largest term in that relation is then removed from the full library, and the procedure is repeated to find additional empirical relations contained in the library. *G_n_* represents the *n*th column of the matrix *G*.

Last, a sparse regression algorithm illustrated in Fig. 3 is applied to each coefficient (Eq. 2). This iterative algorithm finds one or more approximate sparse solutions **c**, which correspond to parsimonious yet quantitatively accurate relations between the fields **u** and **n**. In this manner, nine equations were found across six different libraries (see the Supplementary Materials). It is straightforward to show that all of them can be derived from the following set of three fundamental relations: an incompressibility condition

$$\nabla_{i}u_{i}=0$$ (3)

an evolution equation for the director field

$$\partial_{t}n_{i}+c_{1}u_{j}\nabla_{j}n_{i}+c_{2}{\text{Ω}}_{ij}n_{j}+c_{3}{\hat{P}}_{⊥}{\bar{A}}_{ij}n_{j}=0$$ (4)

and a stress balance

$${\bar{A}}_{kl}{\bar{Q}}_{kl}{\bar{Q}}_{ij}+c_{5}{\bar{Q}}_{ij}=0$$ (5)

with the coefficients *c*_1_ = (0.99 ± 0.8%), *c*_2_ = (−0.95 ± 0.7%), *c*_3_ = (−0.95 ± 1%), and *c*_5_ = (−0.56 ± 1%). Here, ${\hat{P}}_{⊥}$ is the projection operator onto the direction normal to **n**, *A_ij_* and Ω*_ij_* represent, respectively, the symmetric and antisymmetric components of the velocity gradient tensor ∇*_i_u_j_*, and the bar denotes the trace-free component of a symmetric tensor. The two terms in Eq. 5 could be interpreted as an anisotropic viscous stress $\sigma_{ij}^{v}=\mu {\bar{Q}}_{ij}{\bar{A}}_{kl}{\bar{Q}}_{kl}$ and an active stress $\sigma_{ij}^{a}=\alpha {\bar{Q}}_{ij}$, with the coefficient *c*_5_ ∝ −α/μ relating the strengths of activity and viscosity. The activity coefficient α is positive (negative) for an extensile (contractile) nematic (*24*, *46*). The coefficient *c*_5_ is indeed negative, suggesting that α > 0, as it should be for the extensile nematic considered here.

**Fig. 4.**
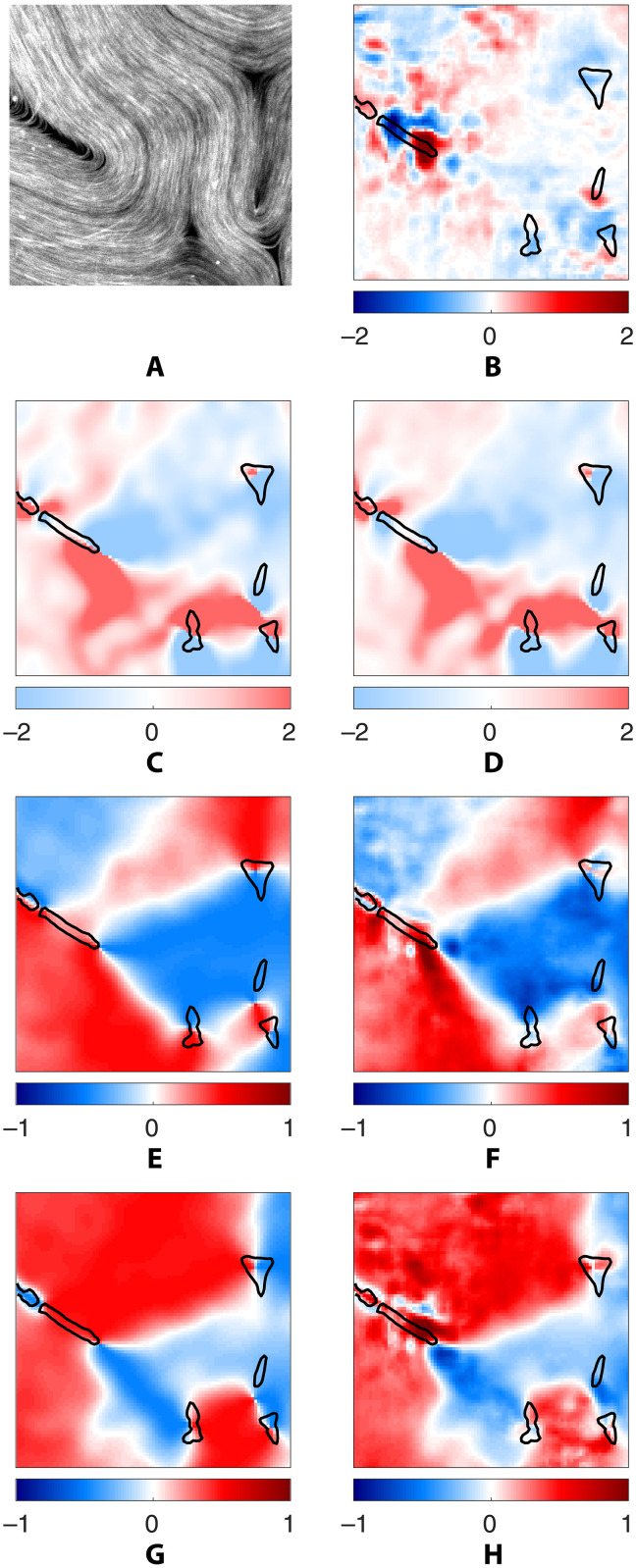
The strong form of the identified relations. Symbolic regression identifies physical relations in weak form. To check the validity of the corresponding PDEs in strong form, we computed each term on the entire spatial domain using finite differences. (**A**) A cropped snapshot of the microtubules, and all other panels correspond to this snapshot. (**B**) Divergence ∇*_i_u_i_* of the interfacial flow. (**C**) Observed angular velocity ∂*_t_*θ = ε*_ij_n_i_∂_t_n_j_* of the microtubules and (**D**) its value reconstructed using the vector relation (4). The remaining panels compare the two components of the active and viscous stresses in arbitrary units: the diagonal component $\sigma_{11}^{a}$ (**E**) and $-\sigma_{11}^{v}$ (**F**) and the off-diagonal component $\sigma_{12}^{a}$ (**G**) and $-\sigma_{12}^{v}$ (**H**). The viscous stress shown in **(**F) and (H) involves spatial derivatives and is therefore much noisier than the active stress shown in (E) and (G). Black solid curves in (B) to (H) correspond to a level set of the number density field ϕ and describe the edges of the regions devoid of microtubules.

Before discussing how these PDEs are related to the existing models of active nematics, let us emphasize that SPIDER ensures that these relations are satisfied in weak form. To check that the strong form of these PDEs is also satisfied, we evaluated and compared various terms at every location in space and time. In particular, the divergence ∇ · **u** for a typical snapshot is shown in Fig. 4B and movie S1. We find that its magnitude is small almost everywhere, consistent with what has been previously reported for the active nematic under consideration (*29*). Regions where the divergence takes large, positive or negative, values are collocated with the regions where the microtubule density is low (ϕ ≈ 0). These regions are excluded from our analysis, so the velocity field can be considered essentially divergence-free where the microtubules are dense (ϕ ≈ 1).

Figure 4 (C and D) and movie S2 compare the field *∂_t_***n** computed directly from the data with that given by Eq. 4. In this case, we find good agreement in the entire domain, not just in the regions with ϕ ≈ 1. Last, Fig. 4 (E to H) and movie S3 compare the active stress $\sigma_{ij}^{a}$ and the viscous stress $\sigma_{ij}^{v}$ that appear in the stress balance relation (5). The two independent components of the (symmetric) stress tensor are shown in Fig. 4 (E to H) and movie S3. Again, we find good agreement in the entire domain. The minor discrepancies that can be seen are a result of insufficient accuracy in the numerical evaluation of the derivatives of the velocity field.

A direct quantitative measure of model accuracy is provided in fig. S1. This figure shows the accuracy of all nine relations (marked by red crosses) found across all the libraries. For example, the incompressibility condition (3) has a relative residual η ≈ 0.04 meaning that the characteristic value of ∇ · **u** is around 4% of the relevant scale (here defined by max*_ij_* ∣∇*_i_u_j_*∣).

## DISCUSSION

Let us now turn to the discussion of the physical insight that the identified relations suggest. First of all, it should be emphasized that we obtained a complete, two-dimensional mathematical description of the problem in the regions with nearly uniform density ϕ of microtubules. This description has the same number of equations (three) as both the Leslie-Ericksen and the Beris-Edwards models, describing the fluid flow and the orientation of the microtubules. Two of these relations, the incompressibility condition (3) and the evolution equation for the director field (4), are the same as in the Leslie-Ericksen model

$$\partial_{t}n+u\;\cdot \nabla n=\text{Ω}n+\lambda {\hat{P}}_{⊥}An+\text{Γ}\frac{\delta }{\delta n}F[n]$$ (6A)

∇⋅u=0 (6B)

$$\partial_{t}u+u\;\cdot \nabla u=\rho^{-1}\nabla \;\cdot \sigma$$ (6C)

since the coefficients *c*_1_ and *c*_2_ are both very close to the expected values of ±1. Furthermore, λ = −*c*_3_ is found to be very close to unity, as expected for thin filaments (*47*). It is notable that no contributions from the free energy *F*[**n**], including those due to elasticity, are identified in the evolution (Eq. 4) or the stress balance relation (Eq. 5). Note that this is not due to masking, as discussed in the Supplementary Materials.

The remaining (Eq. 5) however is rather unexpected. This is a tensor relation representing local balance between active and viscous stresses, not a vector relation representing momentum balance, as is the case in the Leslie-Ericksen and the Beris-Edwards models. Given that we are dealing with a creeping flow, it is hardly unexpected that the terms *∂_t_***u** and **u** · ∇ **u** resulting in acceleration can be ignored, so that Eq. 6C should reduce to the force balance

∇⋅σ=0 (7)

This equation is consistent with the discovered relation (5), provided the stress tensor can be decomposed as σ = σ*^v^* + σ*^a^*, where the viscous stress and active stress were defined previously. Note that, again, no elastic contribution is found, which is consistent with the absence of elastic effects in the evolution (Eq. 4). In our experimental setup, which is characterized by a relatively low density of topological defects, it is mainly the balance of active and viscous stresses that controls the flow (*6*, *22*, *23*) rather than the balance between active and elastic stresses, as is more commonly assumed (*16*, *18*–*21*, *24*–*27*). In addition, we find that the pressure is essentially constant, and thus, we can neglect it as well. Last, the stress tensor σ*^v^* representing viscous effects is highly anisotropic, in contrast to what is commonly assumed. It represents a special case of the more general phenomenological expression

$$\sigma_{ij}^{v}=\nu_{4}A_{ij}+\beta_{1}(A_{kl}n_{k}n_{l})n_{i}n_{j}+\beta_{2}(A_{ik}n_{k}n_{j}+A_{jk}n_{k}n_{i})$$ (8)

proposed by Leslie (*12*). Here β_1_ = ν_1_ − λ(ν_2_ + ν_3_), β_2_ = ν_5_ + λν_2_, and ν_1_ through ν_5_ are the “Leslie viscosities.” Only one term is present (β_1_ ≠ 0), and the other two are either absent or too small to be detected (ν_4_ = β_2_ = 0).

While we refer to the two-dimensional tensors σ*^a^* and σ*^v^* as “stress tensors” in keeping with the convention, it is important to understand that their components do not represent the stress according to its standard definition in three dimensions. For instance, the forces at the interface generated by kinesin (active stress) are balanced by the *xz* and *yz* components of the viscous stresses in the fluid layers above and below the interface which do not appear in our effectively two-dimensional description. Rather, it is more appropriate to think of the relation (5) as a two-dimensional “projection” of the proper stress balance relation in three dimensions. In particular, although σ*^v^* involves spatial derivatives of the interfacial flow velocity rather than the flow velocity itself, it does represent friction stresses (*35*, *36*), as explained in the next section. One can also find there a discussion of the correct physical interpretation of the parameter *c*_5_.

Although the evolution equation for the *Q*-tensor contained in the Beris-Edwards model is not listed among the three fundamental relations (3) to (5), it follows immediately from the evolution (Eq. 4) if the latter is multiplied by *n_j_*. It is also identified via symbolic regression with coefficients very close to those in Eq. 4 (see the Supplementary Materials). In this case, as before, no elastic (or, more generally, free energy) contributions are found. Symbolic regression also identifies several other relations that follow from one of the fundamental relations. For instance, we find a simple relation

$${\bar{Q}}_{ij}{\bar{A}}_{ij}+c_{5}^{\prime }=0$$ (9)

with *c*′_5_ = (−0.55 ± 0.3%) ≈ *c*_5_ that is equivalent to the stress balance (5), although it does not allow an equally intuitive physical interpretation.

Physical constraints play a crucial role in constructing the libraries, and one should be careful to include as much physics as possible and, at the same time, avoid using assumptions that only appear logical but are not physically grounded. Note that two of the three physical relations (3) to (5) describing this system involve no time derivatives and cannot be identified using methods such as Sparse Identification of Nonlinear Dynamics (SINDy) (*48*) which assume their presence. When properly constrained (e.g., respecting all symmetries of the problem without assuming the presence of temporal derivatives), symbolic regression becomes an extremely powerful and general tool for synthesizing scientific knowledge, as the results presented here vividly illustrate.

### The physical nature of the stress balance relation

Equations 5 and 9 describing the fluid flow can be understood from fist principles in the regions where the curvature of the director field **n** is low. Microtubule bundles experience extension in the direction of **n** due to the action of kinesin motors and contraction in the transverse direction favored by depletion. The [adenosine triphosphate (ATP) concentration–dependent] extension rate E is the same as the contraction rate to preserve the mean density of microtubules at the interface. Let us orient the *x* and *y* axes, locally, such that $n=\hat{x}$, so that ${\bar{Q}}_{xx}=-{\bar{Q}}_{yy}=1/2$ and ${\bar{Q}}_{xy}={\bar{Q}}_{yx}=0$. The kinematic condition at the interface containing the microtubules requires a divergence-free interfacial flow to have a velocity

$$u^{i}(x,y)=\hat{x}\partial_{y}\psi -\hat{y}\partial_{x}\psi$$ (10)

where

ψ(x,y)=Exy+f(x)+g(y) (11)

is a two-dimensional stream function and *f*(*x*) and *g*(*y*) are arbitrary functions that represent the mean flow. It is easy to see that the flow (12) satisfies both Eqs. 5 and 9 provided ∂*_xy_*ψ = E = −*c*_5_ > 0. Hence, the microtubule bundles are characterized by a constant extension rate E rather than a constant stress magnitude α. These predictions are fully corroborated by detailed experimental measurements of the flow in a comoving frame (*49*).

**Fig. 5.**
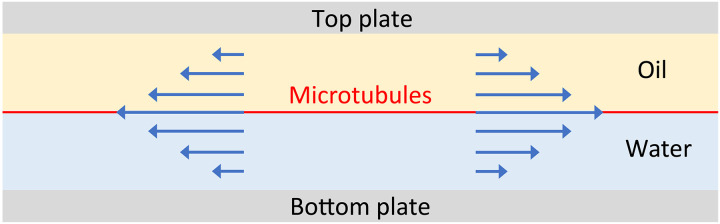
Schematic of the experimental flow cell. Microtubules (shown in red) are confined between a layer of oil and a layer of water. The flow in the *x*-*z* plane for *f*(*x*) = *g*(*y*) = 0 is shown in blue.

The corresponding flow above and below the interface (cf. Fig. 5) can be easily found by assuming its vertical component to vanish everywhere (this assumption is clearly invalid in the regions of nonzero ∇ · **u** near topological defects). Let *z* = 0 denote the interface between the two fluid layers. The corresponding flow field in the bottom fluid layer satisfying the kinematic boundary condition at the interface and the no-slip boundary condition at the bottom *z* = −*h_b_* of the cell is then **u***^b^*(*x*, *y*, *z*) = (1 + *z*/*h_b_*)**u***^i^*(*x*, *y*). Similarly, the flow in the top layer **u***^t^*(*x*, *y*, *z*) = (1 − *z*/*h_t_*)**u***^i^*(*x*, *y*) satisfies the kinematic boundary condition at the interface and the no-slip boundary condition at the top *z* = *h_t_* of the cell. The corresponding viscous stresses at the interface

$$\begin{array}{c} \sigma_{zx}=\mu^{t}\partial_{z}u_{x}^{t}-\mu^{b}\partial_{z}u_{x}^{b}=-\eta u_{x}^{i}=-\eta [Ex+g^{\prime }(y)],\\ \sigma_{zy}=\mu^{t}\partial_{z}u_{y}^{t}-\mu^{b}\partial_{z}u_{y}^{b}=-\eta u_{y}^{i}=-\eta [-Ey+f^{\prime }(x)] \end{array}$$ (12)

are linear in **u***_i_* and represent Rayleigh friction (*35*, *36*). The friction coefficient

$$\eta =\frac{\mu_{t}}{h_{t}}+\frac{\mu_{b}}{h_{b}}$$ (13)

depends on the thicknesses of the two layers and their dynamic viscosities μ*_b_* and μ*_t_*.

We can now obtain a characteristic length scale that balances elastic stresses with viscous stresses caused by the extension of the microtubule bundles. The initial instability leading to an eventual formation of topological defects and controlling their spacing involves buckling of initially straight filaments (*20*, *26*). Let us denote the radius and length of our microtubule bundles as *r* and *L*, respectively. The force exerted by the viscous stresses is given by

$$f_{v}∼Lr\frac{\mu EL}{h}$$ (14)

where *h* and μ describe the layer with the higher ratio μ/*h*, according to the expression (13), and the contribution from the mean flow can be ignored.

Elastic force at the threshold of the buckling instability is given by

$$f_{e}∼\frac{Er^{4}}{L^{2}}$$ (15)

where *E* is the Young’s modulus. Balancing these forces, we find a characteristic length scale

$$L∼r{\left[\frac{h}{r}\frac{E}{\mu E}\right]}^{1/4}$$ (16)

which determines the wavelength of the buckling instability.

Note that this result yields scaling of the length scale with both the activity, described here in terms of the constant extension rate E [which depends on the ATP concentration (*49*)], and the viscosity μ of the fluid which is consistent with experimental observations (*16*, *33*). The length scale increases with the stiffness *Er*^4^ of the microtubules and decreases with the activity (E), as is the case for the standard expression (*9*, *16*, *19*)

$$L∼\sqrt{\frac{K}{\alpha }}$$ (17)

although the functional dependence is clearly different. There are no measurements of *E* and *r* for bundles of microtubules, so we will use the corresponding values for a single microtubule: *r* ∼ 25 nm and *E* ∼ 10^8^ Pa (*50*, *51*). Given the weak dependence of *L* on the ratio *E*/*r*, this should yield a reasonable estimate. In our experiments, μ ∼ 10^−3^ Pa s, *h* ∼ 50 μm, and E ∼ 0.015 s^−1^, so we obtain *L* ∼ 270 μm which is in good agreement with the mean spacing of ∼240 μm between same-charge defects.

### Limitations and future work

It is worth reiterating that the model we have identified does not provide a full description of the dense microtubule suspension at a flat interface. This model only describes regions where the curvature of the microtubules is low and their density ϕ is high and nearly uniform. The dynamics in this system are controlled by the topological defects, the neighborhoods of which have been excluded in our analysis. To properly account for these dynamics, our model has to be generalized to describe the regions around the defects where the curvature is high and the density ϕ varies in both space and time. That generalization has to include an evolution equation for ϕ and incorporate the dependence on ϕ into the remaining governing equations. The former is undoubtedly the continuity equation reflecting mass conservation

$$\partial_{t}\phi +\nabla \cdot j=0$$ (18)

Diffusion of microtubules is negligible due to both confinement to the interface and their large size, hence the flux is likely dominated by advection (*45*),** j **= ϕ**u**, although curvature corrections (*19*) are possible.

The dimensional version of the models (3) to (5) contains only one parameter *c*_5_ which defines a time scale. It does not contain any parameters that can be used to define a length scale. The absence of elastic stresses in our model suggests that interaction between topological defects is mediated by the flow in the two fluid layers when the mean defect separation *L* is large compared with the layer thickness *h*. In our experiment, *L* ≈ 240 μm is larger than *h* = 50 μm. Our results, however, do not exclude the possibility that elastic effects might play a role in the high-curvature regions, requiring generalization of the model. Equation 4 does not appear to need any modification, as it describes the evolution of the director field quite accurately in regions even with low ϕ, as illustrated in Fig. 4 (C and D) and the corresponding movie S2. In addition, both relations describing the fluid flow have to be generalized.

We also emphasize that there is no apriori physical reason for the interfacial fluid flow to be divergence-free everywhere in two dimensions, although the data suggest that the incompressibility (Eq. 3) is satisfied away from the defects. As illustrated in Fig. 4 (A and B) and movie S2, ∇ · **u** is large and positive in the neighborhood of topological defects with +1/2 charge where ϕ changes between low and high values. The regions with ∇ · **u** large and negative are also collocated with the regions where ϕ varies between low and high values but represents past locations of these moving topological defects. In contrast, the flow is found to be essentially divergence-free in the neighborhood of topological defects with −1/2 charge.

Positive divergence is only found in high-curvature regions, suggesting that elastic effects play a key role in creating the defects and pushing the microtubules apart, lowering the density ϕ. There is experimental evidence supporting this role (*17*). We find the divergence to be negative in regions where the curvature is low and the density ϕ is below unity, suggesting that depletion interaction plays an important role. Therefore, one might expect to see two additional dimensional parameters characterizing, respectively, the stiffness of the microtubule bundles and the depletion interaction in the generalized model. In particular, the stiffness parameter can be used to define a characteristic length scale. Exponential distributions of vortex areas at high densities of topological defects suggest the presence of a length scale which also depends on activity (*16*) and viscosity (*33*).

The stress balance (Eq. 5) should also be generalized. In regions without microtubules, where ϕ = 0, the active stresses are expected to vanish, while the viscous stresses are expected to become isotropic, with the latter balanced by the pressure gradient. Regions where the pressure gradients are nonnegligible likely correspond to locations where there is a strong flow toward or away from the interface associated with the large (positive or negative) values of ∇ · **u**. The largest discrepancy between the active and viscous stresses is localized to those regions as well, as illustrated in Fig. 4 (E to H) and movie S2.

**Fig. 6.**
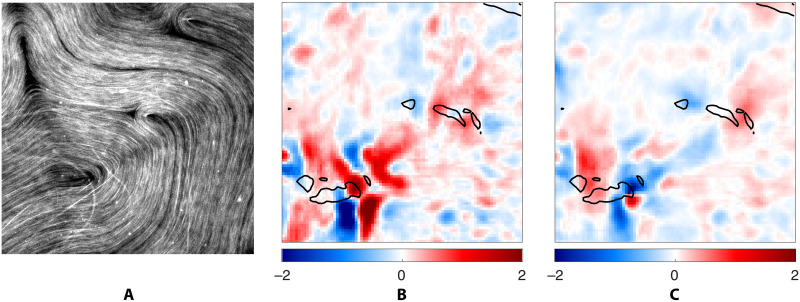
Microtubule alignment and the accuracy of the identified relations. (**A**) Three noticeable out-of-plane microtubule bundles are misaligned with the rest of the microtubules at the bottom left of the image. (**B**) Divergence of the flow is the largest in the regions of misalignment. (**C**) The error (residual) of the scalar form (9) of the stress balance is also the largest there.

Last, let us revisit the assumption of the local order in the average microtubule orientation that underlies the validity of the hydrodynamic description of this system. While the vast majority of microtubules are oriented in the same direction (*S* = 1), there are rare exceptions. An example is shown in Fig. 6A which features several microtubule bundles that are misaligned with the rest. In regions where microtubules cross, their orientation cannot be described by a continuous field and the hydrodynamic description breaks down. This is illustrated in Fig. 6 (B and C) and movies S1 and S3, which show that the error in both the incompressibility condition (3) and the stress balance (5) is the largest in the region where misaligned microtubules are found. This ultimately reflects that there is a three-dimensional aspect of these microtubule suspensions that is often neglected.

It is straightforward to extend our analysis to include additional physical fields such as the microtubule density and kinesin or ATP concentration. The main challenge is the capability to measure and/or reconstruct the corresponding quantities in experiment in parallel with the director and flow fields. Additional fields need to be similarly well-resolved in space and time, so that the corresponding derivatives and integrals can be computed with reasonable accuracy.

## METHODS

### Experimental setup and data acquisition

We conduct our experiments on the microtubule-kinesin active nematic system pioneered in (*8*). The long rod-like microtubules are bundled together via depletion interactions and are driven out of equilibrium by the action of kinesin-streptavidin motor protein complexes, which are units that induce relative motion using ATP as the energy source. Depletion forces also aid in driving the microtubule bundles to form bundles to the oil-water fluid interface, where they execute self-sustained bending and buckling instabilities. The system is extensile, which means that active stresses cause the microtubule bundles to extend in length and contract in width.

To investigate the dynamics of defects in two-dimensional flat space, we prepare the active nematic in a flow-cell setup where the entire pool of ingredients is confined in a two-dimensional sealed cell roughly 10 cm^2^ in area and 100 μm in thickness. The lower surface of the cell is subjected to hydrophobic treatment (using Aquapel) and the upper surface to hydrophilic treatment (using polyacrylamide coating) to enhance wetting by the respective fluid phases. A fluorinated oil (HFE-7500 with surfactant E2K0660) forms the oil-phase, and the active microtubule suspension forms the water-phase. We obtained purified tubulin monomers and kinesin-streptavidin motor protein complexes from the Dogic Group at Brandeis University (*8*, *30*). The polymerization of tubulin to microtubules is performed in our lab before mixing with other biomaterials as per the protocols described in previous works (*8*, *29*, *30*, *52*). The final active mix has 20% microtubules by volume aided with 144μM ATP. The entire flow cell is sealed by epoxy resin and centrifuged at 1000 rpm to accelerate the depletion mechanism to the interface.

We use confocal fluorescence microscopy for visualization. The microtubules are labeled with Alexa Flour 647 dye and illuminated at 633 nm; the excitation and emission peaks are at 651 and 667 nm, respectively. After sample preparation and centrifugation, we wait for 15 to 20 min to allow for uniform depletion and then image at a constant frame rate until the activity ceases. Typically, the microtubules stay active for 6+ hours. Imaging is done using 10× and 20× objectives to focus on regions with area on the order of square millimeters away from the edges of the flow cell. The imaging process results in a time series of 8-bit grayscale images, which are stored as the raw data (*29*).

### Data processing

Two fields are extracted directly from experimental data: the flow velocity u and the nematic director field **n**. The video analyzed has *O*(1000) frames with 512 × 512 resolution and side length of 484.35 μm, collected at a frame rate of 0.88 s^−1^. The flow field *u* is extracted with particle image velocimetry performed with LaVision DaVis 10.1. The resulting flow field is only resolved on a 128 by 128 mesh, and all other fields are restricted to this resolution.

The director field **n** is extracted using coherence-enhancing diffusion filtering (CEDF) (*53*). CEDF determines the direction along which the spatial intensity variation is the smallest, which corresponds to local average alignment of the microtubules. This allows constructing the nematic tensor order parameter ${\bar{Q}}_{ij}$, which, upon diagonalization, yields the nematic director (**n**). A detailed description of the image analysis technique can be found in (*54*). While this method reliably finds **n** relatively far from defects where microtubules are dense, the low microtubule density near the defects prevents resolving the director field in these regions, even when carefully using the blurring schemes inherent in our CEDF methodologies. This can be seen in Fig. 1.

The microtubule density ϕ can also be extracted from experimental images as a function of blurred intensity. Far from defects, the microtubules can be reasonably assumed to be of uniform density. In the neighborhood of defects, the density is discontinuous; it vanishes close to defects where the director field becomes undefined. We restrict our study to the former regions, characterized by negligible density fluctuations. We also ignore the scalar order parameter *S* which describes the local alignment of the microtubules. Far from defects, *S* = 1 as the microtubules are all well-aligned with rare exceptions.

Both **u** and **n** fields are smoothed with a moving least squares multivariate fit, and any necessary derivatives are computed with second-order centered differences. A good choice of units is helpful for model discovery, and we choose a characteristic length and time scale so that mean velocity and vorticity are both unity, 〈∣**u**∣〉 = 〈∣ω∣〉 = 1. In these units, the experimental image sequence has dimensions of *L_x_* × *L_y_* × *L_t_* = 5 × 5 × 17.

### Model libraries

Locality and smoothness imply that a physical relation between the two fields, **u** and **n**, can be written as a superposition (1) of a number of terms *F^r^*, each constructed from **u**, **n**, and/or their spatial and temporal derivatives, i.e., that relation has the form of a PDE. For instance, every relation in the Leslie-Ericksen model (6) has just such a form. For relations involving more than one term, all terms should transform in the same way under every operation in the symmetry group describing the problem (*55*), which includes rotations around the vertical axis and nematic symmetry **n** → −**n**. Hence, terms with different transformation properties are grouped into separate libraries {*F^r^*}.

The rotation symmetry implies that every term in a library has to transform as a tensor of a specific rank (we only consider tensors of rank 0 through 2). The nematic symmetry implies that every term in a library must involve either even or odd powers of **n**. An arbitrary rank 2 tensor can be decomposed into a symmetric and antisymmetric part. The symmetric part can be further decomposed into a traceless component and the trace, with the latter transforming as a scalar. Therefore, without loss of generality, the rank 2 tensor library can be split into two parts: symmetric traceless (denoted with a bar) and antisymmetric (denoted with a tilde).

All the libraries considered in this study are summarized in Table 1 with the terms contained in various libraries listed in the Supplementary Materials. Subscripts denote the tensor indices, and superscripts denote the hyperparameters associated with regression, e.g., the index of the term in the library.

**Table 1.** The summary of the model libraries and their symmetry properties.

Libraries	Even powers of n	Odd powers of n
Rank 0 tensor (scalar)	*F^r^*	${\hat{F}}^{r}$
Rank 1 tensor (vector)	$F_{i}^{r}$	${\hat{F}}_{i}^{r}$
Symmetric traceless rank 2 tensor	${\bar{F}}_{ij}^{r}$	Not studied
Antisymmetric rank 2 tensor	${\tilde{F}}_{ij}^{r}$	Not studied

### Weak formulation

Once a library has been constructed, the corresponding PDE (1) is converted to an overdetermined system of linear algebraic equations *G***c** = 0 for the unknown coefficients **c** = (*c*_1_, *c*_2_, ⋯) following (*42*). This is accomplished by multiplying every term by weight functions *w_k_* and integrating the result over a rectangular spatiotemporal subdomain *V_l_*, with each combination of *k* and *l* defining one or more rows of the feature matrix *G*. Weak form of the PDEs allows SPIDER to deal with noise levels as high as 100% (*55*).

The weight functions *w_k_* are constructed as a product of three components: (i) an envelope (1 − *x*^2^)^τ^(1 − *y*^2^)^τ^(1 − *t*^2^)^τ^ which vanishes, along with τ − 1 derivatives, on the boundary of subdomain *V_l_* to eliminate the boundary terms left after integration by parts; (ii) a modulation term which is taken to be one of $\left\{1,\cos(\pi x-\theta_{x}^{k}),\cos(\pi y-\theta_{y}^{k}),\cos(\pi t-\theta_{t}^{k})\right\}$ with arbitrary phases $\theta_{m}^{k}$, and (iii) a mask ψ which excludes unreliable data. Note that, in the above expressions, *x*, *y*, and *t* have been shifted and scaled such that *V_l_* becomes a cube [−1,1] × [−1,1] × [−1,1]. The choice of τ is determined by the highest-order derivative that appears in the PDE (1); here, we take τ = 4 (*41*). The smooth mask ψ vanishes in regions where the density of microtubules is low and/or their curvature is high and approaches unity far from those regions. It is constructed by successively smoothing the Boolean field ψ_0_, which vanishes when the image intensity is below some threshold or derivatives of **n** are above some threshold and is unity otherwise.

After performing the integration by parts to move as many derivatives as possible from the library term containing noisy data onto a smooth weight function (see the Supplementary Materials for details), each integral is evaluated numerically using the trapezoidal rule (*41*). The subdomains *V_l_* are chosen to include sufficiently many grid points in every direction to ensure reasonable accuracy of numerical quadrature (54 by 54 by 65 grid points) and are centered at uniformly distributed random points of the spatiotemporal domain describing the dataset to ensure that the data are well sampled and to avoid linear dependence.

### Sparse regression

Once the feature matrix *G* has been constructed, we look for a sparse solution **c** that corresponds to a parsimonious physical relation that balances simplicity and accuracy. Simplicity can be measured in a number of ways; here, we take it to be determined by the number of nonzero coefficients *c_r_*. The accuracy can be quantified by a properly normalized residual; we choose to use the 2-norm of the residual for the weak form of the relation, η = Ξ^−1^ ∥*Gc*∥_2_. For relations involving multiple terms, we normalize by the 2-norm of the largest term, Ξ = max*_r_* ∥*c_r_***G***^r^*∥_2_, where **G***^r^* is the *r*th column of *G*. For single-term relations, we instead use the 2-norm of the corresponding tensor before contraction, i.e., Ξ = ∥∇*u*∥_2_ for the incompressibility condition (3).

Parsimonious relations are identified using sequentially thresholded regression (STR). At each step of this iterative algorithm, we compute *c* as the right singular vector of *G* corresponding to the smallest singular value. This corresponds to the solution of a constrained least squares problem *G^T^G***c** = 0 with the normalization ∥**c**∥_2_ = 1 (*55*). We start with the full library and, at each step, discard the term *F^r^* with the smallest magnitude, *r* = arg min ∥*c_r_***G***^r^*∥_2_. Note that quality (completeness) of a library can be quantified by computing the residual η before any terms are discarded. For data not corrupted by noise, a good library should have η ≪ 1.

After discarding a term from the library, the procedure is repeated, with the residual η*_k_* increasing with every iteration *k* as the number of terms in the model decreases. The iteration is terminated when either there is only one term left or the residual increases by a factor exceeding some threshold, i.e., η_*k*+1_ > γη*_k_*, where we typically take γ = 1.15. This choice is somewhat arbitrary, as most of our results are robust to a choice 1.1 ≤ γ ≤ 1.3. In the latter case, we find a multiterm relation, if the final residual is sufficiently small. In the former case, we have to recompute the residual using the normalization appropriate for single-term relations to decide whether that relation is suitably accurate.

Note that STR is not guaranteed to yield the most accurate sparse relation (of a given complexity) contained in the original library. For relatively simple relations (such as the ones identified in this study) and relatively small libraries, we can verify the results of STR by performing a combinatorial search computing the norm of all the relations containing a given number of terms. To validate a relation with *K* terms contained in a library with *N* terms through combinatorial search requires an order of *N*(*N* − 1)⋯(*N* − *K* + 1) operations, which is a tractable problem for the values of *K* and *N* considered here. For relations with larger *K*, the results of STR can be validated by adding one or more terms from the library that decrease the residual the most. If none of these lower the residual appreciably, the result of STR is considered validated.

Multiple relations, including identities, can coexist in the same library. STR is therefore performed iteratively; the library is pruned by throwing out the most complex term of the previously identified relation. STR generally finds identities with machine precision residuals before physical relations with higher residuals. Relations can be more robustly labeled identities by testing them on random smooth synthetic data. Identities will have low residual independent of whether synthetic of experimental data is used, while physical relations are only found for experimental data. Low-dimensional combinatorial searches can more thoroughly explore the relation space to find both identities and physical relations. We stop searching for new relations once the residual for the full pruned library increases above some threshold, e.g., 0.4.

Last, the coefficients *c_r_* of sparse physical relations are found to vary slightly depending on the number (chosen to be an order of magnitude larger than the size of the library) and location of the integration domains. To quantify the uncertainty in the coefficients, *c_r_* is first identified using the entire feature matrix. The coefficients are then recomputed for the same sparse relation using 100 different samples containing half of the rows in the feature matrix and only the columns corresponding to *c_r_*. The mean and SDs of the distribution define the value and uncertainty, respectively, of the corresponding coefficient.

## Supplementary Materials

### This PDF file includes:

Construction of libraries Nematic-invariant scalar library Nematic-covariant scalar library Nematic-covariant vector library Nematic-invariant vector library Symmetric trace-free tensor library Antisymmetric tensor library Data masking and the elastic effects Figs. S1 and S2 Legends for movies S1 to S4 References

## Notes

View/request a protocol for this paper from *Bio-protocol*.
