# Supplementary material for: Physically informed data-driven modeling of active nematics
Source: Sci Adv. 2023 Jul 5;9(27):eabq6120. doi: 10.1126/sciadv.abq6120 (PMC10321743; doi:10.1126/sciadv.abq6120)
Supplement: Supplementary file 1 — Construction of libraries Nematic-invariant scalar library Nematic-covariant scalar library Nematic-covariant vector library Nematic-invariant vector library Symmetric trace-free tensor library Antisymmetric tensor library Data masking and the elastic effects Figs. S1 and S2 Legends for movies S1 to S4 References [file sciadv.abq6120_sm.pdf]

Science Advances
AAAS

Supplementary Materials for

Physically-informed data-driven modeling of active nematics

Matthew Golden *et al.*

Corresponding author: Matthew Golden, mgolden30@gatech.edu

Sci. Adv. **9**, eabq6120 (2023)
DOI: 10.1126/sciadv.abq6120

The PDF file includes:

Construction of libraries
Nematic-invariant scalar library
Nematic-covariant scalar library
Nematic-covariant vector library
Nematic-invariant vector library
Symmetric trace-free tensor library
Antisymmetric tensor library
Data masking and the elastic effects
Figs. S1 and S2
Legends for movies S1 to S4
References

Other Supplementary Material for this manuscript includes the following:

Movies S1 to S4

Supplemental material

Construction of libraries

Construction of the libraries in an ad-hoc manner is prone to errors, so we developed a systematic procedure. The first step is to construct tensors, up to a certain rank, from the vectors such as $\mathbf{u}$, $\mathbf{n}$, and ∇ and scalars such as 1 and ∂_t. The number of different such tensors quickly grows with the rank, so we use known physics to further constrain what terms can appear. In particular, the flow is slow, so inertia is negligible. Hence we allow $\mathbf{u}$ and ∂_t to appear at most once in any tensor. Elastic and viscous effects are described by terms with two spatial derivatives, so we allow ∇ to appear at most twice in any tensor. Lastly, we do not allow mixed spatiotemporal derivatives to appear. There are no physical constraints on $\mathbf{n}$, so this field can appear an arbitrary number of times. Let us define the fundamental tensors of rank k as $\mathcal{T}_{(k)}$:

$$\begin{aligned}
&\mathcal{T}_{(0)} \in \{1\},\\
&\mathcal{T}_{(1)} \in \{\mathbf{u},\, \partial_t\mathbf{n},\, \partial_t\mathbf{u}\} \cup \{\mathbf{n}\mathcal{T}_{(0)}\}\\
&\mathcal{T}_{(2)} \in \{\mathbf{u}\partial_t\mathbf{n},\, \nabla\mathbf{n},\, \nabla\mathbf{u}\} \cup \{\mathbf{n}\mathcal{T}_{(1)}\}\\
&\mathcal{T}_{(3)} \in \{\mathbf{u}\nabla\mathbf{n},\, \nabla\nabla\mathbf{n},\, \nabla\nabla\mathbf{u}\} \cup \{\mathbf{n}\mathcal{T}_{(2)}\}\\
&\mathcal{T}_{(4)} \in \{(\nabla\mathbf{n})(\nabla\mathbf{n}),\, (\nabla\mathbf{u})(\nabla\mathbf{n}),\, \mathbf{u}\nabla\nabla\mathbf{n}\} \cup \{\mathbf{n}\mathcal{T}_{(3)}\}\\
&\mathcal{T}_{(5)} \in \{\mathbf{u}(\nabla\mathbf{n})(\nabla\mathbf{n})\} \cup \{\mathbf{n}\mathcal{T}_{(4)}\}\\
&\mathcal{T}_{(k)} \in \{\mathbf{n}\mathcal{T}_{(k-1)}\},\ \text{for } k > 5
\end{aligned} \tag{S1}$$

The normalization $\mathbf{n}^2 = 1$ constrains the derivatives of $\mathbf{n}$: $n_i\nabla_j n_i = 0$ and $n_i\partial_t n_i = 0$. A stronger consequence is that the gradient tensor $\nabla\mathbf{n}$ is reducible [56]: $\nabla_i n_j = -n_i b_j + s(\delta_{ij} - n_i n_j)$, where $s = \nabla\cdot\mathbf{n}$ is the splay scalar and $\mathbf{b} = -\mathbf{n}\cdot\nabla\mathbf{n}$ is the bend vector. The splay s is nematic-covariant, while b_i is nematic-invariant and $n_i b_i = 0$. This can be used to significantly reduce the number and complexity of the tensors of a given rank. Let us next define the reduced fundamental tensors of rank k, $\mathcal{R}_{(k)}$:

$$\begin{aligned}
&\mathcal{R}_{(0)} \in \{1,\, s,\, s^2\}\\
&\mathcal{R}_{(1)} \in \{\mathbf{u},\, \partial_t\mathbf{n},\, \partial_t\mathbf{u},\, \mathbf{b},\, s\mathbf{u},\, \nabla s,\, s\mathbf{b},\, s^2\mathbf{u}\} \cup \{\mathbf{n}\mathcal{R}_{(0)}\}\\
&\mathcal{R}_{(2)} \in \{\mathbf{u}\partial_t\mathbf{n},\, \nabla\mathbf{u},\, \mathbf{u}\mathbf{b},\, \nabla\mathbf{b},\, \mathbf{b}\mathbf{b},\, s\mathbf{u}\mathbf{b},\, s\nabla\mathbf{u},\, \mathbf{u}\nabla s\} \cup \{\mathbf{n}\mathcal{R}_{(1)}\}\\
&\mathcal{R}_{(3)} \in \{\nabla\nabla\mathbf{u},\, \mathbf{u}\mathbf{b}\mathbf{b},\, \mathbf{b}\nabla\mathbf{u},\, \mathbf{u}\nabla\mathbf{b}\} \cup \{\mathbf{n}\mathcal{R}_{(2)}\}\\
&\mathcal{R}_{(k)} \in \{\mathbf{n}\mathcal{R}_{(k-1)}\} \text{ for } k > 3.
\end{aligned} \tag{S2}$$

It is the contractions of these reduced tensors which make up our libraries. This reduction does not remove all identities from the library, but it eliminates many.

Nematic-invariant scalar library

There are nine nematic-invariant scalars that can be obtained from even-rank reduced fundamantal tensors with the same nematic symmetry listed in (S2):

$$F^r \in \{1,\, s^2,\, n_i u_i s,\, \nabla_i(n_i s),\, \nabla_i u_i,\, \bar{Q}_{ij}\bar{A}_{ij},\, u_i b_i,\, \mathbf{b}^2,\, \nabla_i b_i\} \tag{S3}$$

This library contains a single identity

$$\nabla_i(n_i s + b_i) = 0 \tag{S4}$$

which can be used to prune its last term. These library terms are well-behaved everywhere, and so they can be integrated without any complications yielding the following entries in the feature matrix

$$G^{rkl} = \int_{V_l} w_k F^r\, dV. \tag{S5}$$

Two parsimonious physical relations are identified via symbolic regression, the incompressibility condition (3) and a relation (9) between the director and flow fields with the relative residuals of $\eta = 0.03$ and $\eta = 0.08$,

respectively. Figures S1**(A)** and S1**(B)** illustrate how the residual varies with the number K of terms retained in the relation. Note that the coefficient c_1 has units of inverse time. Its magnitude is $c_1 = O(1)$, which is consistent with our choice of units.

Figure S1**(A)** shows that there is a version of the incompressibility condition involving 5 terms that has an even lower residual ($\eta = 0.02$) than the one-term relation. However, given that our library is missing terms which incorporate the crucial dependence on the microtubule (microtubule) density ϕ (as discussed in the main text), it is rather pointless to look for a physical interpretation of this more general relation.

Nematic-covariant scalar library

There are 18 nematic-covariant scalars that can be constructed from even-rank reduced fundamental tensors with the same nematic symmetry:

$$\tilde{F}^r \in \{s,\, n_i u_i,\, n_i \partial_t u_i,\, u_i \partial_t n_i,\, n_i u_i s^2,\, s u_i b_i,\, s\nabla_i u_i,\, s\bar{Q}_{ij}\bar{A}_{ij},\, u_i n_i n_j \nabla_j s,\, u_i \nabla_i s,$$
$$n_i n_j n_k \nabla_i \nabla_j u_k,\, n_i \nabla^2 u_i,\, n_i \nabla_i \nabla_j u_j,\, u_i n_i \mathbf{b}^2,\, b_i n_j \bar{A}_{ij},\, b_j n_i \Omega_{ij},\, u_i n_j \nabla_j b_i,\, n_i u_i \nabla_j b_j\}, \qquad \text{(S6)}$$

where the last term can be eliminated using the identity (S4). The nematic-covariant scalar library $\{\hat{F}^r\}$ involves discontinuous fields. Director field changes sign at "branch cuts" connecting the topological singularities, causing problems with evaluating derivatives. To restore continuity, all terms are multiplied by $\mathbf{n}$, making every term a vector. The two components of this vector are treated separately, effectively doubling the number of rows of G (one for $i = 1$ and another for $i = 2$):

$$\hat{G}_i^{rkl} = \int_{V_l} w_k n_i \hat{F}^r \, dV. \qquad \text{(S7)}$$

The most accurate parsimonious physical relation identified via symbolic regression contains two terms:

$$s\left[\bar{Q}_{ij}\bar{A}_{ij} + c_5^{(2)}\right] = 0, \qquad \text{(S8)}$$

where $c_5^{(2)} = -0.57 \pm 1\%$. This relation follows from (9) as long as $c_5^{(2)} = c_5'$ and, indeed, the values of $c_5^{(2)}$ and c_5' are found to be very close. Figure ()**(C)** shows how the residual varies during regression. The inclusions of the additional factor $s = \nabla \cdot \mathbf{n}$ increases the residual to $\eta = 0.23$, almost three-fold compared with (9), which highlights the importance of the quality of the data-processing algorithm (here the one that extracts $\mathbf{n}$ from the images), especially for relations containing derivatives.

Nematic-covariant vector library

The nematic-covariant vector library is expected to include an angular momentum balance relation describing the evolution of the director field such as (6a) and hence contains the term $\partial_t \mathbf{n}$. Since $|\mathbf{n}| = 1$, this time derivative should be orthogonal to $\mathbf{n}$, and without loss of generality, we can restrict our attention to vectors orthogonal to $\mathbf{n}$ that can be constructed from the reduced fundamental odd-rank tensors with the same nematic symmetry:

$$\tilde{F}_i^r \in \{\partial_t n_i,\, s u_i,\, \nabla_i s,\, s b_i,\, \bar{A}_{ij} n_j,\, \Omega_{ij} n_j,\, u_j \nabla_j n_i,\, n_j \nabla_j b_i\}, \qquad \text{(S9)}$$

where the term $n_j u_j b_i$ has been replaced by its more familiar form $u_j \nabla_j n_i$. We can exclude the component of every term $\hat{\mathbf{F}}^r$ along $\mathbf{n}$ and eliminate the discontinuities in the director field by considering the z component of the vector product $\hat{\mathbf{F}}^r \times \mathbf{n}$ or, in the index notation:

$$G^{rkl} = \int_{V_l} \varepsilon_{ij} w_k n_i \hat{F}_j^r \, dV. \qquad \text{(S10)}$$

No identities are found in this library. Symbolic regression identifies one parsimonious physical relation (4). This relation is formally equivalent to the evolution equation (6a) of the Leslie-Ericksen model (sans the elastic contribution $\Gamma\mathbf{h}$) with the coefficients c_r that are very close to ± 1. The relative residual $\eta = 0.08$ is quite low and comparable to that of equation (9). Figure S1**(D)** shows how it varies during the regression.

Nematic-invariant vector library

The nematic-invariant vector library would be expected to include a momentum balance relation such as (6c), which contains divergences of various stresses. The library constructed from the reduced fundamental odd-rank tensors with the same nematic symmetry

$$\begin{aligned}F_i^r \in \{&sn_i,\, u_i,\, (n_ju_j)n_i,\, \partial_t u_i,\, n_in_j\partial_t u_j,\, b_i,\, u_is^2,\, n_in_ju_js^2,\, n_ju_j\partial_t n_i,\, n_iu_j\partial_t n_j,\, sn_ju_jb_i,\\ &su_jb_jn_i,\, n_is\bar{Q}_{jk}\bar{A}_{jk},\, sn_j\nabla_i u_j,\, sn_j\nabla_j u_i,\, sn_i\nabla_j u_j,\, n_i(n_ju_j)(n_k\nabla_k s),\, u_in_j\nabla_j s,\\ &n_ju_j\nabla_i s,\, n_jn_k\nabla_j\nabla_k u_i,\, n_jn_k\nabla_i\nabla_j u_k,\, u_i\mathbf{b}^2,\, \mathbf{b}^2u_jn_jn_i,\, u_jb_jb_i,\, b_i\bar{A}_{jk}\bar{Q}_{jk},\, b_i\nabla_j u_j,\\ &b_j\nabla_j u_i,\, b_j\nabla_i u_j,\, n_ju_jn_k\nabla_k b_i,\, u_i\nabla_j b_j,\, u_j\nabla_i b_j,\, u_j\nabla_j b_i\}\end{aligned} \tag{S11}$$

will not include stress tensors which involve two spatial derivatives, such as the elastic stress tensor. To get around this, we extended this library by explicitly including higher-order terms of the form $\nabla_j F^r_{ij}$, where

$$\begin{aligned}F^r_{ij} \in \{&\bar{Q}_{ij},\, s^2\bar{Q}_{ij},\, su_in_j,\, su_jn_i,\, su_kn_k\bar{Q}_{ij},\, (n_k\nabla_k s)\bar{Q}_{ij},\, n_i\nabla_j s,\, n_j\nabla_i s,\, n_isb_j,\, n_jsb_i,\\ &(\nabla_k u_k)\bar{Q}_{ij},\, (\bar{A}_{kl}\bar{Q}_{kl})\bar{Q}_{ij},\, (\nabla_i u_k)n_kn_j,\, (\nabla_j u_k)n_kn_i,\, \bar{A}_{ij},\, \Omega_{ij},\, (u_kb_k)\bar{Q}_{ij},\, u_kn_kb_in_j,\\ &u_kn_kb_jn_i,\, u_ib_j,\, u_jb_i,\, (\nabla_k b_k)\bar{Q}_{ij},\, (n_k\nabla_k b_i)n_j,\, (n_k\nabla_k b_j)n_i,\, \nabla_i b_j,\, \nabla_j b_i,\, \mathbf{b}^2\bar{Q}_{ij},\, b_ib_j\}\end{aligned} \tag{S12}$$

is a library of nematic-invariant rank-2 tensors. Note that these tensors include the elastic, viscous and active stresses present in the Leslie-Eriksen model, but not the pressure. Since pressure is a latent field that we have no data for, we should ignore both terms of the form $\nabla_i p$ in (S11) and diagonal stress tensors of the form $p\delta_{ij}$ in (S12), where p is any scalar field.. This can be achieved by considering the z component of the vector products $F^r \times \nabla w_k$ in the weak formulation. In the index notation, this corresponds to

$$G^{rkl} = \int_{V_l} \varepsilon_{ij}(\nabla_i w_k)F^r_j\, dV. \tag{S13}$$

There are a number of identities in this library that will not be listed explicitly. Symbolic regression identified two parsimonious relations:

$$(\bar{A}_{kl}\bar{Q}_{kl} + c_5^{(2)})sn_i + \nabla_i p = 0, \tag{S14}$$

$$\nabla_k\left[(\bar{A}_{lm}\bar{Q}_{lm} + c_5^{(3)})\bar{Q}_{ik} + \delta_{ik}p\right] = 0, \tag{S15}$$

where $c_5^{(2)} = -0.57 \pm 1\%$ and $c_5^{(3)} = -0.59 \pm 1\%$. Both relations follow from (9) for $c_5^{(2)} = c_5'$ and $c^{(3)} = c_5'$ and, indeed, these coefficients are found to be rather close. The relative residual of these relations $\eta = 0.28$ and $\eta = 0.38$, respectively, are notably higher then the residual for relation (9), which is not surprising due to the presence of several additional derivatives in the weak form of these relations. The variation of the residuals during regression is shown in Figures S1**(E)** and S1**(F)** .

Symmetric trace-free tensor library

It is not always convenient to use a bar to denote the symmetric trace-free part of a tensor. Let us introduce an alternative notation $T_{(ij)} = \frac{1}{2}(T_{ij} + T_{ji} - \delta_{ij}T_{kk})$. We can construct the library of nematic-invariant symmetric trace-free tensors using even-rank reduced fundamental tensors:

$$\begin{aligned}\bar{F}^r_{ij} \in \{&\bar{Q}_{ij},\, s^2\bar{Q}_{ij},\, \partial_t\bar{Q}_{ij},\, su_{(i}n_{j)},\, su_kn_k\bar{Q}_{ij},\, (n_k\nabla_k s)\bar{Q}_{ij},\, n_{(i}\nabla_{j)}s,\, sn_{(i}b_{j)},\\ &\nabla_k u_k\bar{Q}_{ij},\, (\bar{A}_{kl}\bar{Q}_{kl})\bar{Q}_{ij},\, \bar{A}_{ij},\, (u_kb_k)\bar{Q}_{ij},\, u_kn_kb_{(i}n_{j)},\, (\nabla_k b_k)\bar{Q}_{ij},\\ &(n_k\nabla_k b_{(i})n_{j)},\, (\nabla_i b_j)',\, \mathbf{b}^2\bar{Q}_{(ij)},\, \bar{A}_{k(i}\bar{Q}_{j)k},\, b_{(i}b_{j)},\, u_{(i}b_{j)}\}.\end{aligned} \tag{S16}$$

This library can be handled in the same way as the nematic-invariant scalar library:

$$\bar{G}^{rkl}_{ij} = \int_{V_l} w_k\bar{F}^r_{ij}\, dV, \tag{S17}$$

$$\tag{S18}$$

Note that symmetric trace-free tensors have two independent components $ij = 11$ and 12, doubling the number of rows in G.

Three identities appear in this library.

$$\bar{A}_{k(i}\bar{Q}_{j)k} = 0,$$
$$b_{(i}b_{j)} + \mathbf{b}^2 Q_{(ij)} = 0,$$
$$u_k \nabla_k Q_{(ij)} - 2su_{(i}n_{j)} + 2su_k n_k Q_{(ij)} + 2u_k b_k \bar{Q}_{(ij)} + 2u_{(i}b_{j)} = 0. \tag{S19}$$

We use these to discard the last two library terms. Two physical relations are found in this library, the stress balance relation (5) and an evolution equation for the orientation tensor

$$\partial_t Q_{ij} + c_1' u_k \nabla_k \bar{Q}_{ij} + c_2'(\Omega_{ik}\bar{Q}_{kj} - \bar{Q}_{ik}\Omega_{kj}) + c_3' \bar{A}_{ij} + c_4' Q_{ij}(\bar{A}_{kl}\bar{Q}_{kl}) = 0, \tag{S20}$$

where $c_1' = 1 \pm 0.1\%$, $c_2' = -0.96 \pm 0.1\%$, $c_3' = -1.02 \pm 0.1\%$, and $c_4' = 2.05 \pm 0.1\%$. These relations have low residuals $\eta = 0.1$ and $\eta = 0.09$, respectively. For comparison, the tensor balance between $\bar{A}_{ij}$ and $\bar{Q}_{ij}$ proposed in Ref. [22] has a much higher residual $\eta = 0.67$. The variation of the residuals during regression is shown Figure S1(g-h). Note that relation (S20) corresponds to the nematodynamics equation of the Beris-Edwards model and can be derived by multiplying the evolution equation (4) by n_j. This gives the following correspondence between the coefficients: $c_1' = c_1$, $c_2' = c_2$, $c_3' = c_3$, and $c_4' = -2c_3$.

Using a lower STR threshold $\gamma = 1.1$, the residual of the relation (5) can be decreased slightly (about 10%) by including a term A_{ij} representing isotropic viscous contribution. The corresponding coefficient is quite small (-0.02), which suggests that the isotropic contribution to viscosity is negligible in the regions of high density of microtubules. On the other hand, this term is expected to be dominant in the regions with the low density of microtubules. Hence, in general, one should expect the viscous stresses to include both contributions ($\bar{Q}_{kl}A_{kl}\bar{Q}_{ij}$ and A_{ij}) with the coefficients dependent on the microtubule density ϕ. The coefficients are also expected to depend on the shape of the nematic units, similar to the tumbling parameter λ. It should be possible to derive these coefficients from first principles by solving for the flow above and below the interface with appropriate boundary conditions.

Antisymmetric tensor library

Let $T_{[ij]} = \frac{1}{2}(T_{ij} - T_{ji})$ be the antisymmetric part of a rank-2 tensor. Again, we can construct the library of antisymmetric tensors using even-rank reduced fundamental tensors:

$$\tilde{F}^r_{ij} \in \{n_{[i}\partial_t n_{j]},\ su_{[i}n_{j]},\ n_{[i}\nabla_{j]}s,\ sn_{[i}b_{j]},\ n_k n_{[i}\nabla_{j]}u_k,\ \Omega_{ij},\ u_k n_k b_{[i}n_{j]},\ u_{[i}b_{j]},$$
$$n_{[i|}n_k\nabla_k b_{|j]},\ \nabla_{[i}b_{j]}\}. \tag{S21}$$

This library can be handled in the same way as the nematic-invariant scalar library:

$$\tilde{G}^{rkl}_{ij} = \int_{V_l} w_k \tilde{F}^r_{ij}\, dV. \tag{S22}$$

We find a single identity

$$u_k n_k b_{[i}n_{j]} + u_{[i}b_{j]} = 0 \tag{S23}$$

and a single physical relation

$$n_{[j}\partial_t n_{i]} + c_1'' su_{[i}n_{j]} + c_2'' u_{[i}b_{j]} + c_3''\Omega_{ij} + c_4'' n_k n_{[i}\nabla_{j]}u_k = 0, \tag{S24}$$

where $c_1'' = 1.03 \pm 0.2\%$, $c_2'' = 1.04 \pm 0.9\%$, $c_3'' = -0.98 \pm 0.5\%$, and $c_4'' = -1.00 \pm 0.7\%$. This relation can also be derived from the evolution equation (4) provided $c_1'' = -c_2'' = c_1$, $2c_3'' + c_4'' = c_2$, and $c_4'' = -c_3$. The relative residual for relation (S24) is $\eta = 0.05$, making it the most accurate representation of $\partial_t \mathbf{n}$ found. The variation of the residual during regression is shown in Figure S1(**I**) .

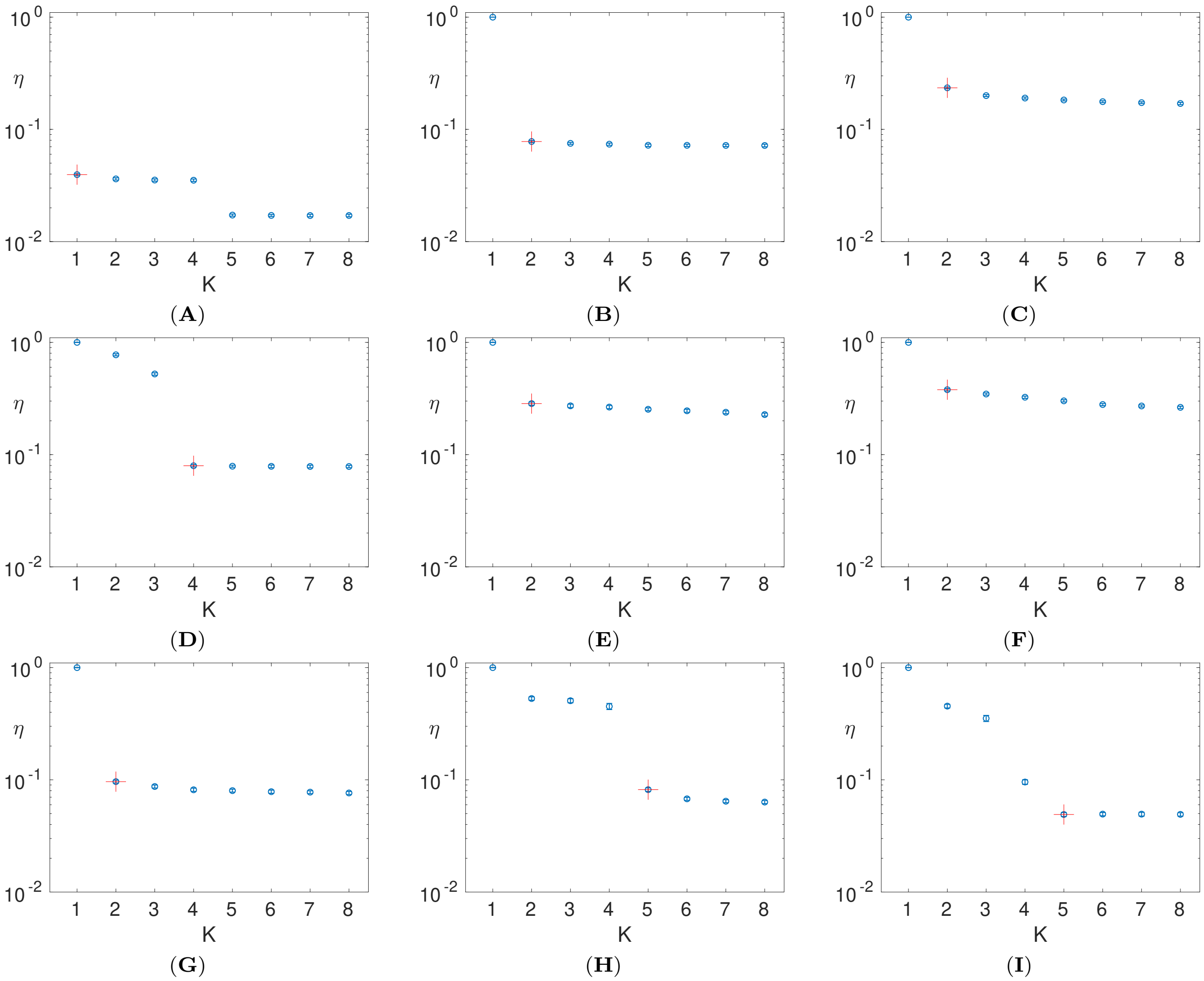

Figure S1 : **The relative residual η as a function of K, the number of terms in the relation.** The plots corresponding to discovered equations are **(A)** equation (3), **(B)** equation (9), **(C)** equation (S8), **(D)** equation (4), **(E)** equation (S14), **(F)** equation (S15), **(G)** equation (5), **(H)** equation (S20), and **(I)** equation (S24). The red cross indicates the corresponding parsimonious relation. These plots were generated by first identifying a parsimonious relations with iterative STR and then adding (removing) terms that decrease (increase) the residual the most (least).

Data masking and the elastic effects

Elastic effects should be the strongest in the neighborhood of topological defects, where the curvature of the director field and hence the deformation of microtubules is the highest. Masking used in the weak formulation eliminates the data from these regions from our analysis. We address here whether this might lead to regression eliminating any terms involving spatial derivatives of $\mathbf{n}$, including the elastic contributions that might otherwise appear in equations (4) and (5).

In practice, masking retains data with sufficiently high curvature to reliably identify elastic effects, if there were any. To see this, let us compare the distribution of the absolute values of the bend $\mathbf{b}$ and splay s for the data before and after masking. Figure S2 shows the probability distribution functions (PDF) describing each of these quantities computed using data that have been nondimensionalized as discussed in the Methods section. While masking does affect the PDF, it retains data with sufficiently high values of both $|\mathbf{b}|$ and $|s|$ for all elastic terms to retain their order of magnitude after the mask has been applied.

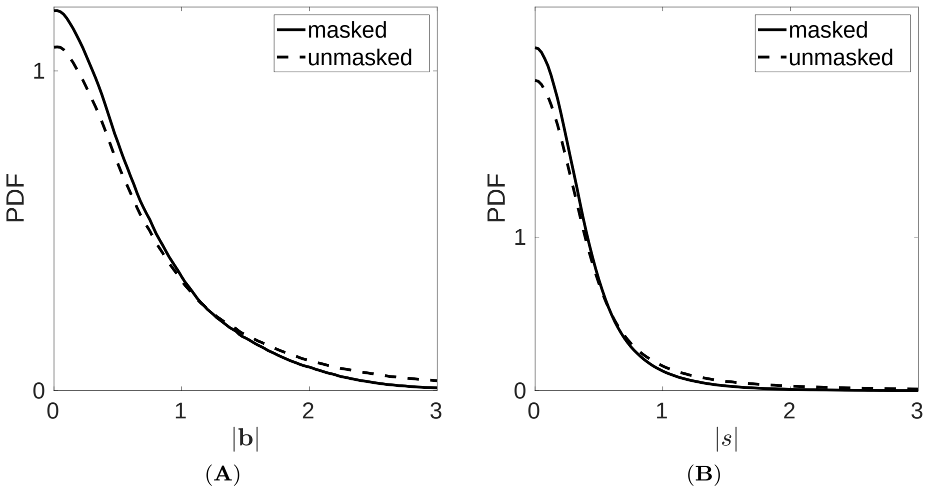

Figure S2 : **The effect of masking on the curvature.** Shown are the PDF of the magnitude of the bend vector $\mathbf{b} = -(\mathbf{n} \cdot \nabla)\mathbf{n}$ **(A)** and the splay scalar $s = \nabla \cdot \mathbf{n}$ **(B)** . For the purpose of computing the PDF, unmasked data were chosen to correspond to regions with $\psi > 0.1$.

Supplemental Movie Captions

Movie S1: **The divergence of the interfacial flow.** The divergence of the experimental flow field (left) is compared with the corresponding experimental images (right). The scale of the color bar is arbitrary, and the black contours correspond to boundaries of low-density regions.

Movie S2: **The angular velocity of the microtubules**. The left panel shows the angular velocity $\partial_t\theta$ obtained by finite differencing the data, and the right panel shows the reconstruction using the right-hand side of the discovered PDE (3). The scale of the color bar is arbitrary, and the black contours correspond to boundaries of low-density regions.

Movie S3: **Comparison of the two terms in the effective stress balance equation (4).** A diagonal component of the active stress tensor is shown on the left and the same component of the viscous stress tensor is shown on the right. The scale of the color bar is arbitrary, and the black contours correspond to boundaries of low-density regions.

Movie S4: **The mask used in analyzing the data.** The mask used to filter out data from low-density regions in our analysis (left) is compared with the corresponding experimental image (right). The mask (red) is overlaid with the director field (black arrows).

REFERENCES AND NOTES

1. J. Toner, Y. Tu, Flocks, herds, and schools: A quantitative theory of flocking. *Phys. Rev. E* **58**, 4828–4858 (1998).
2. J. Toner, Y. Tu, S. Ramaswamy, Hydrodynamics and phases of flocks. *Ann. Phys. Rehabil. Med.* **318**, 170–244 (2005).
3. S. Ramaswamy, The mechanics and statistics of active matter. *Annu. Rev. Condens. Matter Phys.* **1**, 323–345 (2010).
4. M. C. Marchetti, J. F. Joanny, S. Ramaswamy, T. B. Liverpool, J. Prost, M. Rao, R. A. Simha, Hydrodynamics of soft active matter. *Rev. Mod. Phys.* **85**, 1143–1189 (2013).
5. F. Jülicher, S. W. Grill, G. Salbreux, Hydrodynamic theory of active matter. *Rep. Prog. Phys.* **81**, 076601 (2018).
6. A. Doostmohammadi, J. Ignés-Mullol, J. M. Yeomans, F. Sagués, Active nematics. *Nat. Commun.* **9**, 3246 (2018).
7. V. Narayan, S. Ramaswamy, N. Menon, Long-lived giant number fluctuations in a swarming granular nematic. *Science* **317**, 105–108 (2007).
8. T. Sanchez, D. T. N. Chen, S. J. DeCamp, M. Heymann, Z. Dogic, Spontaneous motion in hierarchically assembled active matter. *Nature* **491**, 431–434 (2012).
9. N. Kumar, R. Zhang, J. J. De Pablo, M. L. Gardel, Tunable structure and dynamics of active liquid crystals. *Sci. Adv.* **4**, eaat7779 (2018).
10. H. Li, X.-Q. Shi, M. Huang, X. Chen, M. Xiao, C. Liu, H. Chaté, H. P. Zhang, Data-driven quantitative modeling of bacterial active nematics. *Proc. Natl. Acad. Sci.* **116**, 777–785, (2019).
11. J. L. Ericksen, Conservation laws for liquid crystals. *Trans. Soc. Rheol.* **5**, 23–34 (1961).
12. F. M. Leslie, Some constitutive equations for liquid crystals. *Arch. Ration. Mech. Anal.* **28**, 265–283, (1968).
13. P.-G. De Gennes, J. Prost, *The physics of liquid crystals*. No. 83, Oxford University Press, 1993.
14. A. N. Beris, B. J. Edwards, *Thermodynamics of flowing systems: with internal microstructure*. No. 36, Oxford University Press on Demand, 1994.
15. K.-T. Wu, J. B. Hishamunda, D. T. N. Chen, S. J. DeCamp, Y.-W. Chang, A. Fernández-Nieves, S. Fraden, Z. Dogic, Transition from turbulent to coherent flows in confined three-dimensional active fluids. *Science* **355**, eaal1979 (2017).

16. L. M. Lemma, S. J. DeCamp, Z. You, L. Giomi, Z. Dogic, Statistical properties of autonomous flows in 2D active nematics. *Soft Matter* **15**, 3264–3272, (2019).
17. A. Opathalage, M. M. Norton, M. P. N. Juniper, B. Langeslay, S. A. Aghvami, S. Fraden, Z. Dogic, Self-organized dynamics and the transition to turbulence of confined active nematics. *Proc. Natl. Acad. Sci. U.S.A.* **116**, 4788–4797, (2019).
18. S. P. Thampi, R. Golestanian, J. M. Yeomans, Velocity correlations in an active nematic. *Phys. Rev. Lett.* **111**, 118101 (2013).
19. L. Giomi, M. J. Bowick, P. Mishra, R. Sknepnek, M. Cristina Marchetti, Defect dynamics in active nematics. *Phil. Trans. R. Soc. A* **372**, 20130365 (2014).
20. S. P. Thampi, R. Golestanian, J. M. Yeomans, Instabilities and topological defects in active nematics. *EPL* **105**, 18001 (2014).
21. L. Giomi, Geometry and topology of turbulence in active nematics. *Phys. Rev. X* **5**, 031003 (2015).
22. S. P. Thampi, A. Doostmohammadi, R. Golestanian, J. M. Yeomans, Intrinsic free energy in active nematics. *EPL* **112**, 28004 (2015).
23. S. P. Thampi, J. M. Yeomans, Active turbulence in active nematics. *Eur. Phys. J. Spec. Top.* **225**, 651–662 (2016).
24. R. Green, J. Toner, V. Vitelli, Geometry of thresholdless active flow in nematic microfluidics. *Phys. Rev. Fluids* **2**, 104201 (2017).
25. A. Doostmohammadi, T. N. Shendruk, K. Thijssen, J. M. Yeomans, Onset of meso-scale turbulence in active nematics. *Nat. Commun.* **8**, 15326 (2017).
26. B. Martínez-Prat, J. Ignés-Mullol, J. Casademunt, F. Sagués, Selection mechanism at the onset of active turbulence. *Nat. Phys.* **15**, 362–366 (2019).
27. R. Alert, J.-F. Joanny, J. Casademunt, Universal scaling of active nematic turbulence. *Nat. Phys.* **16**, 682–688 (2020).
28. D. J. G. Pearce, P. W. Ellis, A. Fernandez-Nieves, L. Giomi, Geometrical control of active turbulence in curved topographies. *Phys. Rev. Lett.* **122**, 168002 (2019).
29. D. J. G. Pearce, J. Nambisan, P. W. Ellis, A. Fernandez-Nieves, L. Giomi, Orientational correlations in active and passive nematic defects. *Phys. Rev. Lett.* **127**, 197801 (2021).
30. S. J. DeCamp, G. S. Redner, A. Baskaran, M. F. Hagan, Z. Dogic, Orientational order of motile defects in active nematics. *Nat. Mater.* **14**, 1110–1115 (2015).

31. R. Zhang, Y. Zhou, M. Rahimi, J. J. de Pablo, Dynamic structure of active nematic shells. *Nat. Commun.* **7**, 13483 (2016).
32. C. Blanch-Mercader, V. Yashunsky, S. Garcia, G. Duclos, L. Giomi, P. Silberzan, Turbulent dynamics of epithelial cell cultures. *Phys. Rev. Lett.* **120**, 208101 2018.
33. B. Martínez-Prat, R. Alert, F. Meng, J. Ignés-Mullol, J.-F. Joanny, J. Casademunt, R. Golestanian, F. Sagués, Scaling regimes of active turbulence with external dissipation. *Phys. Rev. X* **11**, 031065 (2021).
34. J. Colen, M. Han, R. Zhang, S. A. Redford, L. M. Lemma, L. Morgan, P. V. Ruijgrok, R. Adkins, Z. Bryant, Z. Dogic, M. L. Gardel, J. J. de Pablo, V. Vitelli, Machine learning active-nematic hydrodynamics. *Proc. Natl. Acad. Sci.* **118**, e2016708118 (2021).
35. S. P. Thampi, R. Golestanian, J. M. Yeomans, Active nematic materials with substrate friction. *Phys. Rev. E* **90**, 062307 (2014).
36. A. Doostmohammadi, M. F. Adamer, S. P. Thampi, J. M. Yeomans, Stabilization of active matter by flow-vortex lattices and defect ordering. *Nat. Commun.* **7**, 10557 (2016).
37. M. Bär, R. Hegger, H. Kantz, Fitting partial differential equations to space-time dynamics. *Phys. Rev. E* **59**, 337 (1999).
38. D. Xu, O. Khanmohamadi, Spatiotemporal system reconstruction using fourier spectral operators and structure selection techniques. *Chaos* **18**, 043122 (2008).
39. S. H. Rudy, S. L. Brunton, J. L. Proctor, J. N. Kutz, Data-driven discovery of partial differential equations. *Sci. Adv.* **3**, e1602614 (2017).
40. H. Schaeffer, Learning partial differential equations via data discovery and sparse optimization. *Proc. Math. Phys. Eng. Sci.* **473**, 20160446 (2017).
41. D. R. Gurevich, P. A. Reinbold, R. O. Grigoriev, Robust and optimal sparse regression for nonlinear pde models. *Chaos* **29**, 103113 (2019).
42. P. A. Reinbold, D. R. Gurevich, R. O. Grigoriev, Using noisy or incomplete data to discover models of spatiotemporal dynamics. *Phys. Rev. E* **101**, 010203 (2020).
43. P. A. K. Reinbold, L. M. Kageorge, M. F. Schatz, R. O. Grigoriev, Robust learning from noisy, incomplete, high-dimensional experimental data via physically constrained symbolic regression. *Nat. Commun.* **12**, 3219 (2021).
44. Z. Zhou, C. Joshi, R. Liu, M. M. Norton, L. Lemma, Z. Dogic, M. F. Hagan, S. Fraden, P. Hong, Machine learning forecasting of active nematics. *Soft Matter* **17**, 738–747 (2021).

45. K. A. Mitchell, A. J. Tan, J. Arteaga, L. S. Hirst, Fractal generation in a two-dimensional active-nematic fluid. *Chaos* **31**, 073125 (2021).

46. R. Aditi Simha, S. Ramaswamy, Hydrodynamic fluctuations and instabilities in ordered suspensions of self-propelled particles. *Phys. Rev. Lett.* **89**, 058101 (2002).

47. R. G. Larson, *The structure and rheology of complex fluids*, vol. 150. Oxford University Press New York, 1999.

48. S. L. Brunton, J. L. Proctor, J. N. Kutz, Discovering governing equations from data by sparse identification of nonlinear dynamical systems. *Proc. Natl. Acad. Sci. U.S.A.* **113**, 3932–3937 (2016).

49. L. M. Lemma, M. M. Norton, A. M. Tayar, S. J. DeCamp, S. A. Aghvami, S. Fraden, M. F. Hagan, Z. Dogic, Multiscale microtubule dynamics in active nematics. *Phys. Rev. Lett.* **127**, 148001 (2021).

50. A. Kis, S. Kasas, B. Babić, A. J. Kulik, W. Benoît, G. A. Briggs, C. Schönenberger, S. Catsicas, L. Forró, Nanomechanics of microtubules. *Phys. Rev. Lett.* **89**, 248101 (2002).

51. J. Zhang, S. Guan, Tensile properties of microtubules: A study by nonlinear molecular structural mechanics modelling. *Phys. Lett. A* **384**, 126674 (2020).

52. P. W. Ellis, D. J. G. Pearce, Y.-W. Chang, G. Goldsztein, L. Giomi, A. Fernandez-Nieves, Curvature-induced defect unbinding and dynamics in active nematic toroids. *Nat. Phys.* **14**, 85–90 (2018).

53. J. Weickert, Coherence-enhancing diffusion filtering. *Int. J. Comput. Vis.* **31**, 111–127 (1999).

54. P. W. Ellis, J. Nambisan, A. Fernandez-Nieves, Coherence-enhanced diffusion filtering applied to partially-ordered fluids. *Mol. Phys.* **118**, e1725167 (2020).

55. D. R. Gurevich, P. A. Reinbold, R. O. Grigoriev, Learning fluid physics from highly turbulent data using sparse physics-informed discovery of empirical relations (spider). arXiv preprint arXiv:2105.00048, 2021.

56. J. V. Selinger, Interpretation of saddle-splay and the oseen-frank free energy in liquid crystals. *Liq. Cryst. Rev.* **6**, 129–142 (2018).
